# CREB Ameliorates Osteoarthritis Progression Through Regulating Chondrocytes Autophagy *via* the miR-373/METTL3/TFEB Axis

**DOI:** 10.3389/fcell.2021.778941

**Published:** 2022-06-09

**Authors:** Haibin Zhang, Xilei Li, Yusheng Li, Xucheng Yang, Runzhi Liao, Haoyi Wang, Junxiao Yang

**Affiliations:** ^1^ Department of Orthopedics, Xiangya Hospital, Central South University, Changsha, China; ^2^ Department of Anesthesiology, Xiangya Hospital, Central South University, Changsha, China; ^3^ National Clinical Research Center for Geriatric Disorders, Xiangya Hospital, Central South University, Changsha, China

**Keywords:** osteoarthritis, autophagy, CREB, METTL3, miR-373

## Abstract

Osteoarthritis (OA) is a degenerative joint disease characterized by articular cartilage degradation. Dysregulated autophagy is a major cause of OA. However, the underlying mechanism is unclear. Here, we found that the expression of element-binding protein (CREB) was downregulated in both cartilage tissues of OA patients and mouse OA model. In tert-butyl hydroperoxide solution-treated chondrocytes, increased apoptosis and autophagic blockage were attenuated by CREB overexpression. Mechanically, MiR-373 directly targeted the 3′UTR of methyltransferase-like 3 (METTL3) and led to its downregulation. METTL3 epigenetically suppressed TFEB. The upregulation of miR-373 by CREB overexpression induced the release of TFEB from METTL3 and restored the autophagy activity of chondrocytes. Taken together, our study showed that CREB alleviates OA injury through regulating the expression of miR-373, which directly targeted METTL3, and finally relieved TFEB from METTL3-mediated epigenetic suppression. The CREB/miR-373/METTL3/TFEB axis may be used as a potential target for the treatment of OA.

## Highlights

1 CREB is decreased in OA while the regaining of this protein attenuates OA-caused chondrocytes apoptosis and autophagy blockage
2 The expression of miR-373 is maintained by the binding of CREB to its promoter
3 METTL3 expression is suppressed by miR-373, which is necessary for TFEB-mediated autophagy and survival of chondrocytes in OA

## Introduction

Osteoarthritis (OA) is a degenerative joint disease characterized by articular cartilage degradation in synovial joints. Since the cartilage serves as a firm and tenacious pad protecting the ends of long bones at the joint, the destruction of the tissues at this site always leads to physical disability to a different degree (Wang et al., 2011). It has long been believed that “wear and tear” is the etiological factor for the initiation and progression of this condition (Aigner et al., 2004) since the OA lesion is often localized to weight-bearing cartilage or sites of injury (Dieppe and Kirwan, 1994). With more in-depth knowledge about OA, OA is now considered a heterogeneous group of disorders with various pathogenic factors, but all lead to similar patterns of cartilage degeneration.

The main structural protein of cartilage is type II collagen, which provides a basic framework that receives stabilization from other collagen types and non-collagenous proteins, including but not limited to Collagens type IX, X, XI, aggrecan, decorin, biglycan, and fibromodulin (Roughley and Lee, 1994; Sophia Fox et al., 2009). As the most dominant cell type in cartilage, chondrocytes are in full responsibility in the generation of the complex cartilage architecture and the regulation of the biochemical composition (Woo et al., 1989; Alford and Cole, 2005). Changes in the chemical, mechanical, or cytokine exposure in the articular environment arouse responses from chondrocytes, which is mainly presented as the altered balance between catabolic and anabolic activities in the cartilage maintenance (Martel-Pelletier et al., 2008; Guo et al., 2017). Based on the understanding of the cartilage cellularity supported by chondrocytes, it is now generally accepted that the OA progression is an outcome of imbalanced chondrocyte activity conducted by abnormal biomechanical, biochemical, and genetic factors (Hering, 1999; Liu et al., 2016).

Autophagy is an essential homeostatic process by which cells break down their components to maintain survival, differentiation, development, or homeostasis in various tissues or organs including cartilage (Mizushima et al., 2008). It has been proved that the dysregulation of several key autophagy genes has a causative relation to OA pathogenesis (Sasaki et al., 2012; Caramés et al., 2013). In OA samples, the expression of the most upstream autophagy inducer, Unc-51–like kinase 1 (ULK1), autophagy structural and functional factor, microtubule-associated protein 1 light chain 3 (LC3B), and key autophagy regulator Beclin1 are all reduced. More than that, inhibition of autophagy would cause OA-like gene expression changes, while artificial induction of autophagy reduced MMP13 and ADAMTS5 expression induced by interleukin (IL)-1β, a major pro-inflammatory cytokine implicated in OA (Kapoor et al., 2011; Zhang et al., 2015). Many potential OA therapies are working as autophagy inducers to promote the survival and activity of chondrocytes, such as rapamycin (Pal et al., 2015). By inducing lysosome biogenesis and promoting autophagosome formation and lysosome fusion, TFEB turns to be a master regulator of the autophagic flux (Sardiello et al., 2009; Settembre et al., 2011). It has been proved that TFEB overexpression protects chondrocytes *in vitro* from tert-butyl hydroperoxide solution (TBHP)-induced lysosome dysregulation and attenuates the surgically induced OA by promoting autophagy in cartilage tissues (Zheng et al., 2018). However, how TFEB is regulated in the related process is still far from clear.

As a great variety of stimuli can modulate autophagy, it is not surprising that a growing number of signaling pathways have been shown to control this process, like tyrosine kinase receptors, casein kinase II (Holen et al., 1993), MAP kinases (Haussinger et al., 1999; Ogier-Denis et al., 2000), and calcium (Gordon et al., 1993), and cAMP response element-binding protein (CREB) pathway is a novel member in this family. CREB is a well-known transcription factor that plays a positive role in cell survival and proliferation while protecting cells from harmful stimuli and apoptosis (Teich et al., 2015; Saura and Cardinaux, 2017). Recently, its role in autophagy regulation has been proved, showing that CREB inhibition directly leads to blockage of autophagy activation (Chong et al., 2018). More than that, this transcriptional activator upregulates autophagy genes, including ATG7, ULK1, and TFEB (Seok et al., 2014). Inspiringly, accumulating evidence implies that the CREB pathway is also engaged in the regulation of chondrocytes activity and cartilage maintenance (Bui et al., 2012). However, the underlying mechanism is far from being well studied.

In the present study, we identified that CREB directly binds to micro-RNA-373 to activate its expression in chondrocytes. The increased miRNA-373 dramatically represses the amount of its target, methyltransferase-like 3 (METTL3), an essential epigenetic regulator, which releases TFEB from the suppression of METTL3-conducted epigenetic modification. This discovery may not only bring a new angle of view to the understanding of OA pathology but also provide several new targets for the potential treatment of this disorder.

## Materials and Methods

### Human Cartilage Tissues

This study was performed with the approval of the Human Ethics Review Committees of The Xiangya Hospital of Central South University. For material collection, informed consent was obtained in writing from each subject or family of the donor. OA cartilage samples were obtained from OA knee joints of patients within 4 h after surgery. The diagnosis of OA was based on the criteria for knee OA of the American College of Rheumatology. Control cartilage samples were obtained from nonarthritic knee joints from traumatic amputee donors. The donors had no known history of joint disease, and the normality of the joint was confirmed macroscopically at the time samples were obtained.

### Chondrocytes Isolation and Culture

To isolate the chondrocytes, the cartilage shavings were minced into small chips (0.5–1 mm) and then incubated with recombinant collagenase class II (300 U/ml) and thermolysin (1 mg/ml) for 6 h at 37°C, 5% CO_2_. Filter the collagenase/chips solution through a 20-μm nylon filter membrane to obtain dissociated chondrocytes. Centrifuge to collect and wash the chondrocytes. Then, chondrocytes were seeded in a cell culture plate and cultured in DMEM/F12 supplemented with 10% FBS and 1% antibiotic in 5% CO_2_ at 37 °C. To establish an OA model *in vitro*, chondrocytes were treated with oxidative stress inducer tert-butyl hydroperoxide (0, 6.25, 12.5, and 25 μM, TBHP) (Sigma, 458139) for 24 h.

### Animal and DMM Model Establishment

All experimental protocols were approved by the Institutional Animal Care and Use Committee of The Xiangya Hospital of Central South University. Male 6-week-old BALB/c wild-type mice were purchased from the Laboratory Animal Center of the Institute of Genetics in Beijing and maintained under specific pathogen-free conditions with 12/12-h light/dark cycles, at 25°C, with fodder (Rat & Mouse Maintenance Diet 1022, HFK Bio-tech, China) and water *ad libitum*.

For DMM surgery, mice were anesthetized with 300 mg/kg intraperitoneal Tribromoethanol and the knees were prepared for aseptic surgery. Buprenorphine (Buprenex^®^, Reckitt and Coleman Products, Kingston-Upon-Hull, United Kingdom) was provided peri-operatively at 0.09 mg/kg. Surgery was operated under direct visualization and strictly followed the details described in a previous study (Glasson, Blanchet, and Morris 2007).

### Immunohistochemistry

Cartilage tissues were fixed in 10% formalin for 24 h and embedded in paraffin. Then, blocks were sectioned into 5 μm thick for immunohistochemical (IHC) examination. Sections on slides were dewaxed in xylene and rehydrated in ethanol gradient. Antigen recovery was accomplished in boiling 0.01% sodium citrate buffer. Primary antibody, CREB (ProteinTech, 12208-1-AP), was diluted in antibody dilution buffer (3% BSA, 1% normal donkey serum in Tris-buffered saline) for incubation. Slides were then incubated with the primary antibody overnight at 4°C. Slides were processed using the biotin-streptavidin HRP detection system (SP-9002, ZSGB-BIO, Beijing, China) followed by 3,3′-diaminobenzidine (DAB) staining, with counter-staining by hematoxylin. Slides were sealed in neutral resins with coverslips for subsequent detection. Images of the tissue sections were captured by a Zeiss imaging system (Zeiss, Germany).

### Autophagic Flux Assay

The assay was operated according to a protocol from the manufacturer for the application of LC3B antibody (NB100-2220, Novus Bio, Colorado United States). In detail, chloroquine diphosphate (CQ) (10 mM) was added to the culture dishes at designated time points to a final concentration of 50 µM and incubated overnight (16 h). Cells were harvested and subjected to immunocyto-fluorescence staining. Dilute primary antibody in 1% BSA in PBS. Incubate samples with 5 μg/ml rabbit anti-LC3B primary antibody at 4°C overnight. After wash, apply an appropriate dilution of fluorophore-conjugated anti-rabbit secondary antibody in 1% BSA. After wash and counter-staining with DAPI, use a fluorescence microscope to examine and image the cells.

### TUNEL Assay

The assay was accomplished by commercial kit according to the manufacturer’s manual (ab66108, Abcam, Cambridge, United Kingdom). In detail, harvest cells at designated time points and wash with PBS and then spin onto slides. Fix cells with cold formaldehyde for 4 min and then wash with PBS. Add ice-cold 70% ethanol and incubate for 30 min. Wash with wash buffer twice and then incubate with staining solution for 60 min at 37°C. Add rinse buffer wash samples twice. Resuspend cells in DAPI/RNase A solution and incubate for 30 min at room temperature. Then, analyze results under a fluorescence microscope.

### LysoTracker Staining

The staining procedure was accomplished by a LysoTracker™ kit (L7528, Invitrogen, California United States). When cells have reached the desired confluence, remove the medium from the dish and add the prewarmed (37°C) probe-containing medium. Incubate the cells for 1 h. Then, cells were washed with fresh medium and harvested for ICC analysis under a microscope.

### Quantitative Real-Time PCR

RNA from the cartilage tissues or chondrocytes was extracted by TRIZOL Reagent (Invitrogen, Life Technologies, United States). According to the manufacturer’s suggestion, 1.5-fold suggested volume of isopropanol was used to make sure RNA in small size, including miRNA, could be well precipitated. The TRIZOL-extracted RNA samples were then applied to Turbo DNase treatment to remove possible genomic DNA contamination. For mRNA quantification, 1 μg of purified total RNA was used for reverse transcription (Promega Reverse Transcription System) following the manufacturer’s protocol. The iTaq Universal SYBR Green Supermix (Bio-Rad, 1725120) was used for the mRNA qPCR reaction. To quantify miRNA expression change, the TaqMan microRNA assay kit (Applied Biosystems) was used for the reverse transcription and qPCR following the manufacturer’s protocol. qPCR reactions were operated in triplicate and analyzed by the Applied Biosystems QuantStudio 6 Pro real-time PCR system (Life Technologies, CA, United States). The comparative Ct (2^^^-ΔΔCt) method was used to determine the relative gene expression change. GAPDH was used as an endogenous reference for mRNA quantification and U6 was used for miRNA quantification. The primer sequences were as follows: CREB-F: 5′-AGT​TTG​ACG​CGG​TGT​GTT​AC-3′; CREB-R: 5′-TAC​CTG​GGC​TAA​TGT​GGC​AA-3′; miR-373-F: 5′-CGC​GAA​GTG​CTT​CGA​TTT​TG-3′; miR-373-R: 5′-GTG​CAG​GGT​CCG​AGG​T-3’; METTL3-F: 5′-GAG​TGC​ATG​AAA​GCC​AGT​GA-3′; METTL3-R: 5′-CTG​GAA​TCA​CCT​CCG​ACA​CT-3’; TFEB-F: 5′-TGA​TCC​ACT​TCT​GTC​CAC​CA-3′; TFEB-R: 5′-GCA​GGT​GGC​TAC​TTC​ACA​CA-3’; GAPDH-F: 5′-CTG​ACT​TCA​ACA​GCG​ACA​CC-3′; GAPDH-R: 5′-GTG​GTC​CAG​GGG​TCT​TAC​TC-3′; U6- F: 5′-CTC​GCT​TCG​GCA​GCA​CA-3’; U6-R: 5′-AAC​GCT​TCA​CGA​ATT​TGC​GT-3′.

### Western Blotting

The total protein of cartilage tissues or chondrocytes was prepared by homogenizing the tissues in WIP Tissue and Cell lysis solution (CellChip Beijing Biotechnology Company, Beijing, China) following the manufacturer’s instructions. Protein samples (10 μg each) were loaded on 10% SDS–PAGE and transferred to polyvinylidene fluoride membranes (IPVH00010, Millipore, MA, United States). Membranes were incubated overnight at 4°C with corresponding antibodies. The secondary antibody (IRDye 680RD, LI-COR Biosciences, United States) was diluted at 1:10,000 in Tween-Tris-buffered saline. Blottings were visualized by Odyssey CLx Imaging System (LI-COR Biosciences, United States). GAPDH was used as an endogenous reference. The density of the bands was normalized to that of GAPDH.

Antibodies used for blotting in this study are as follows: BAX (Santa Cruz, sc-7480), Bcl-2 (Abcam, ab196495), Cleaved Caspase-3 (Cell Signaling Tech, #9664), CREB (ProteinTech, 12208-1-AP), GAPDH (Abcam, ab9485), LAMP2 (Cell Signaling Tech, #49067), LC3I (Novus Bio, NB100-2331), LC3II antibody (Novus Bio, NB100-2220), METTL3 (Abcam, ab195352), P62 (Cell Signaling Tech, #5114), and TFEB (Cell Signaling Tech, #4240).

### Plasmids, Transfection, and RNA Knockdown

cDNAs of human CREB and METTL3 were generated by PCR and cloned into *Asc*I and *Fse*I sites of the pCS2-CMV expression plasmid. Primer sequence: CREB, forward primer—5′-GGGTTTGGCCGGCCAATGACCATGGAA-3′, reverse primer—5′-GGGTTTGGCGCGCCT TAATCTGAT-3’; METTL3, forward primer—5′-GGGTTTGGCCGGCCATGTCGGACA-3′, reverse primer—5′-GGGTTTGGCGCGCCCTATAAATTC-3’. Transfection of plasmids was performed by using Effectene Transfection Reagent (Qiagen, 301425) according to the manufacturer’s instructions.

Short hairpin RNA targeting CREB (sh-CREB) or scrambled control sequence (sh-NC) were designed and produced by GenePharma (Shanghai, China). Cells were transfected with sh-CREB or sh-NC using Lipofectamine 3000 following the manufacturer’s instructions.

For the miRNA mimics and inhibitor transfection, cultured cells were transiently transfected with miR-373 mimic (GenePharma), miR-373 inhibitor (GenePharma, Shanghai, China), or corresponding negative control (GenePharma, Shanghai, China) using Lipofectamine 3000. The total RNA and protein were extracted for further investigation as indicated.

### Dual-Luciferase Reporter Assay

To verify the interaction and the effect of CREB onto the miR-373 promoter, the luciferase reporter vectors containing the promoter region of miR-373 or its three different mutants were constructed. The mutations were conducted by using the QuikChange II XL Site-Directed Mutagenesis Kit (Agilent). The wild-type or mutant promoter reporter vectors together with CREB-overexpression plasmid were co-transfected into cultured normal chondrocytes, which used empty plasmid as control.

Likewise, to verify the interaction between miR-373 and METTL3, the predicted target site for miR-373 on METTL3 RNA transcripts or its mutant were integrated into the luciferase reporter vector. miR-373 mimics were co-transfected with the reporter vectors into the cells. The reporter gene activity was determined by measuring the luciferase activity using Dual-Luciferase®Reporter Assay System (Promega, E1910). The luciferase activity was normalized with the activity of the internal control enzyme, Renilla luciferase on the same vector, to adjust the transfection efficiency between different groups.

### Quantification of TFEB m6A Modification

Briefly, total RNA was isolated by using TRIzol as mentioned above in the method of RT-qPCR. Total RNA (3 mg) was well mixed with 3 fmol spike-in and equally split for three following processes: m6A/m-RIP, IgG control, and input. m6A/m-RIP and IgG control samples were incubated in parallel with 1 mg of anti-m6A/m antibody (Synaptic Systems, l202 003) or 1 mg of rabbit IgG in immunoprecipitation buffer with RNasin Plus (Promega, N2111). Then, the mixture was incubated with washed Dynabeads M-280 (Thermo Fisher, 11203) to capture the antibody–RNA complexes. The bead-bound antibody–RNA complexes were recovered on a magnetic stand and the RNA was extracted again by TRIzol. The RNA products, including input samples, were reverse-transcribed by following the mentioned method in qRT-PCR. The abundance of TFEB was then quantified by following the method in qRT-PCR. The measurements of m6A-TFEB for each treatment were normalized to their corresponding input amount.

### Chromatin Immunoprecipitation

Briefly, approximately 50 mg of chondrocyte homogenates was first cross-linked with 2 mM disuccinimidyl glutarate (DSG, Thermo Fisher Scientific) and 1 mM MgCl_2_ for 40 min, and second cross-linked with 1% formaldehyde for 10 min at room temperature. Extracted nuclei constituents were sonicated to fragment DNA to approximately 0.5 kb. Fragmented DNA was immunoprecipitated with CREB antibodies (ProteinTech, Rosemont, IL, 12208-1-AP) overnight at 4°C. Protein G beads (Sigma, St. Louis, MO, P3296) were added and rocked for 3 h at 4°C. The beads were washed and reverse-crosslinked in elution buffer overnight at 65°C. DNA was purified by phenol/chloroform. Anti-rabbit IgG antibody (Santa Cruz Biotechnology, Dallas, TX, sc-2027) was used as a negative control. Then, qPCR was conducted to measure following the method mentioned above.

### Experimental Design and Statistical Analyses

All the data were expressed as mean ± SD with each model performed in triplicate. Appropriate statistical significance was chosen based on the experimental strategy using GraphPad Prism. Data were analyzed either by *t*-test or ANOVA. Values of *p* < 0.05 were considered statistically significant.

## Results

### CREB is Dramatically Decreased With the Occurrence of OA

**FIGURE 1:**
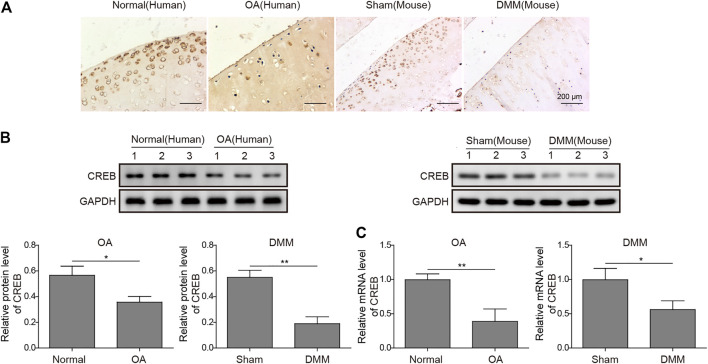
Abnormal expression of CREB in clinic human OA tissues and surgically induced mouse OA model. **(A)** The *in situ* expression changes in human OA samples and mouse-induced OA tissues were examined by IHC. Scale bar = 200 μm. **(B)** The protein level of CREB was determined by Western blotting and then quantified. CREB signal was normalized to GAPDH intensity. **(C)** The expression of CREB mRNA was determined by qRT-PCR. GAPDH was used as the endogenous reference for normalization. *p* < 0.05 was marked as *, *p* < 0.01 was marked as **.

To build up the connection between CREB and OA pathogenesis, we first examined the CREB expression in both normal and OA clinical specimens of humans. As detected by IHC, the CREB staining was clear and perfectly colocalized with the chondrocytes (Figure 1A). In contrast, the chondrocytes in OA samples showed a much weaker CREB signal compared to normal tissues (Figure 1A). To confirm that this was a conservative phenomenon among different species, we then employed one of the most conventional OA mouse models, the DMM model, to examine the CREB expression. Similar to the result in human tissues, CREB was also decreased in DMM mice cartilage tissues. This decrease of CREB was also confirmed at the whole protein and mRNA level. As shown, in both human and mouse samples, the mRNA and protein of CREB were significantly decreased (Figures 1B,C, *p* < 0.01 or *p* < 0.05). These results indicated that the CREB expression level was closely connected to the occurrence of OA. Meanwhile, the reproduction of this phenomenon in the induced OA mouse model also proved its rationality in the study on CREB’s role in OA.

### Complemental CREB Under TBHP Prevents Apoptosis and Autophagic Flux Blockage

**FIGURE 2:**
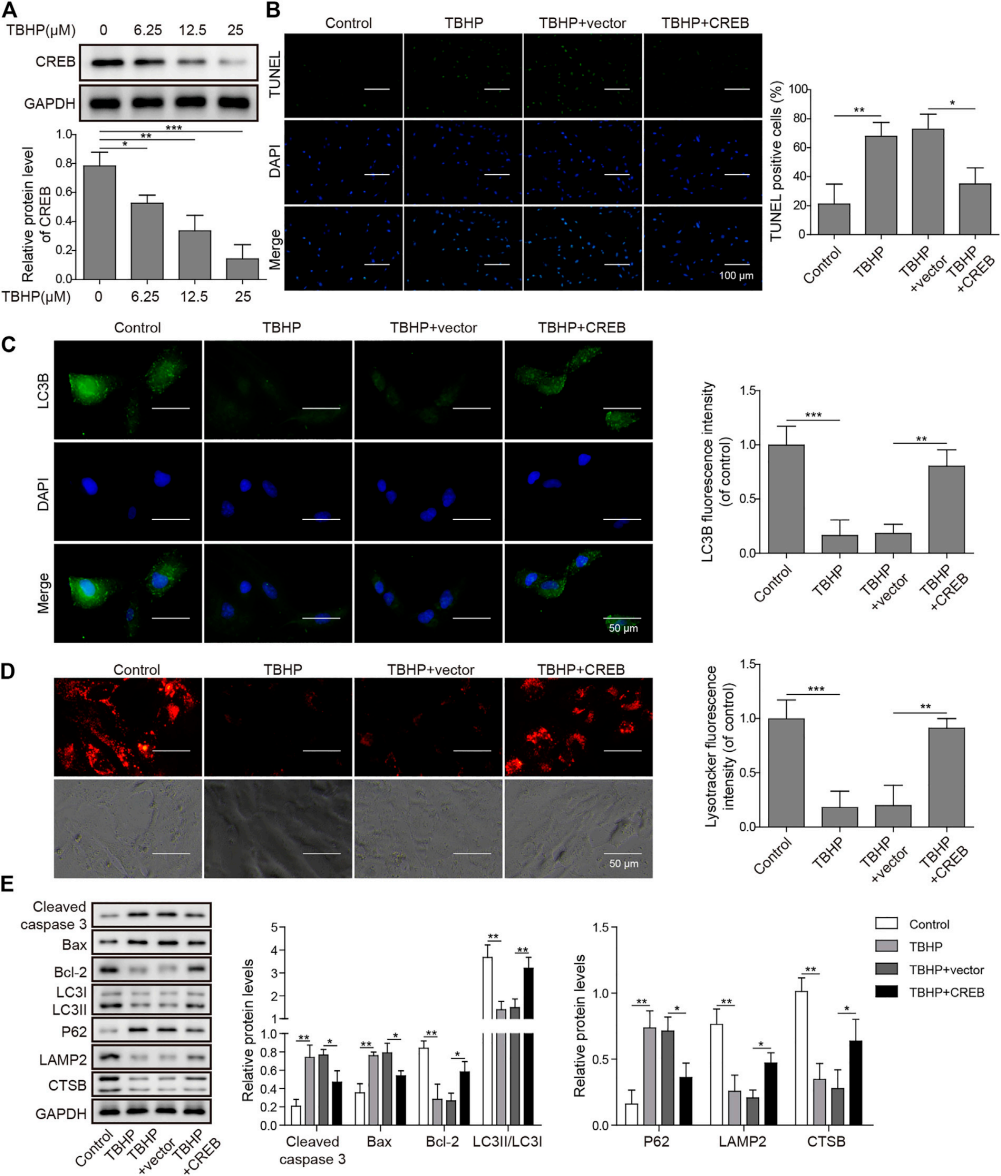
Overexpression of CREB-attenuated TBHP-induced apoptosis and blockage of autophagic flux in cultured human chondrocytes. **(A)** The change in CREB protein under different dosages of TBHP (0, 6.25, 12.5, and 25 μM) was determined by Western blotting and then the intensity of bands was quantified. **(B)** The apoptosis caused by either TBHP alone or together with CREB overexpression was determined by TUNEL assay. The empty vector in the same concentration as CREB-overexpression plasmids was used as a control. TUNEL-positive cells were counted to quantify the outcome of each treatment. **(C)** The autophagic flux under treatments of either TBHP alone or together with CREB overexpression was determined by the immunocyto-fluorescence staining of autophagosome marker LC3B. LC3B fluorescence intensity was measured and data from all groups were normalized to the control group. **(D)** The change of lysosome activity under treatments above was determined by LysoTracker staining. LysoTracker fluorescence intensity was measured and data from all groups were normalized to the control group. **(E)** The impact of the treatments on the essential markers of apoptosis and autolysosome was determined by Western blotting. Quantified intensity of the bands was normalized to GAPDH to generate the column chart. *p* < 0.05 was marked as *, *p* < 0.01 was marked as **, *p* < 0.001 was marked as ***.

To clarify the causative relation between CREB level and OA development, we treated *in vitro* cultured human chondrocytes with TBHP, a widely used OA model *in vitro*. In this model, TBHP steadily releases oxidative stress to mimic the biomechanically induced oxidative stress regulating mechanosensitive signals in chondrocytes (Zheng et al., 2018). Interestingly, CREB was decreased along with the increasing dosage of TBHP. Under the highest concentration of TBHP (25 µM), CREB protein was decreased by 81.7% (Figure 2A, *p* < 0.001). Considering that the CREB/CRE transcriptional pathway is widely regarded as essential protective machinery for cells under oxidative pressure, we presumed that a complemental CREB might help alleviate the TBHP-induced OA phenotype. To prove this idea, a CREB-overexpression vector was constructed and transfected into *in vitro* cultured chondrocytes (CREB group) with the empty vector as a control (vector group). Apoptosis was determined by the TUNEL assay. In the control group, the apoptosis signal was barely detected while there was a marked increase in apoptosis caused by the TBHP treatment (Figure 2B). Vector groups still showed a comparable number of apoptotic cells to TBHP individual treated groups, but CREB groups had much fewer TUNEL positives. The quantitative analysis further supported the cytological result, showing a significant difference between vector and CREB groups (*p* < 0.05). Health cartilage relies on active and well-orchestrated autophagy to effectively remodel the extracellular matrix in the tissues and further prevent chondrocyte death, OA-like changes in gene expression, as well as cartilage degeneration. As shown in the *in situ* autophagic flux assay by LC3B immunocyto-fluorescence staining (Figure 2C), TBHP largely diminished the LC3B fluorescence signal and empty vector transfection of chondrocyte did not affect this decrease. However, with the overexpression of CREB, the level of LC3B was restored almost to the level observed in controls (Figure 2D). Meanwhile, we also examined whether or not lysosomes, as another essential participant in autophagy flux, were under the regulation of CREB. Similar to the condition of autophagosome, LysoTracker-indicated lysosomes were also largely removed under TBHP treatment but recovered with the CREB overexpression. Consistent with these results, CREB overexpression reversed all the TBHP-induced changes of proteins functioning in various categories (Figure 2E). In detail, pro-apoptosis indicators/factors cleaved caspase-3 and Bax, autophagic flux blockage marker P62 (Tang et al., 2017), were promoted by TBHP but the increase was attenuated by CREB overexpression. Apoptosis inhibitor Bcl-2, active autophagy-lysosome pathway markers including LC3-II, lysosome-associated membrane protein-2 (LAMP2), and cystatin B (CSTB) were all decreased by TBHP, while overexpression of CREB rescued their amount. Collectively, the results above imply that the CREB level is positively correlated with the viability and normal function of chondrocytes under oxidant pressure.

### CREB Controls Chondrocyte Apoptosis and Autophagy Through miR-373

**FIGURE 3:**
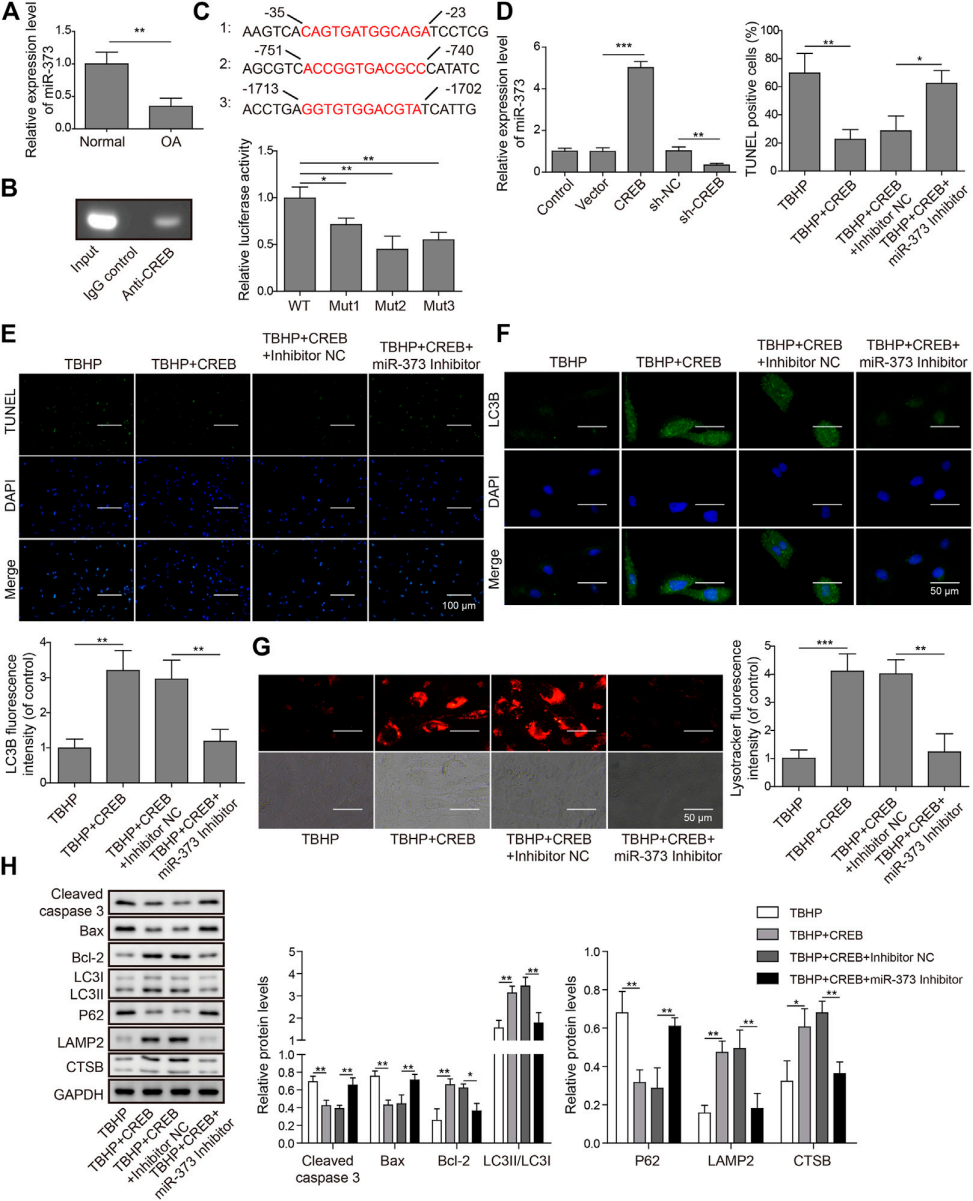
CREB regulated apoptosis and autophagic flux through miR-373. **(A)** The expression of miR-373 in normal and OA human cartilage tissues was determined by qRT-PCR. **(B)** The binding of CREB at the miR-373 promoter was determined by ChIP assay. **(C)** A schematic diagram of CREB binding domain on the miR-373 promoter. The promoting activity of CREB to different mutated miR-373 promoters was measured by luciferase reporter assay. The signal data from each group were normalized to WT to generate the column chart. **(D)** Chondrocytes were transfected with or without CREB-overexpressed vectors (or empty vectors) or sh-CREB (or sh-NC). The expression of miR-373 after transfection was measured by qRT-PCR. **(E)** The effect of miR-373 inhibitor on apoptosis under CREB overexpression was determined by TUNEL assay. The TUNEL-positive cells were numbered to generate the column chart. **(F)** The effect of miR-373 inhibitor on the CREB-promoted autophagic flux in the TBHP-induced *in vitro* OA model was determined by the immunocyto-fluorescence staining of autophagosome marker LC3B. To generate the column chart, LC3B fluorescence intensity was measured and data from all groups were normalized to the TBHP group. **(G)** The change of lysosome activity in the same model was determined by LysoTracker staining. LysoTracker fluorescence intensity was measured and data from all groups were normalized to the TBHP group. **(H)** The impact of the treatments on the essential regulators or markers of apoptosis and autolysosome was determined by Western blotting. The quantified intensity of the bands was normalized to GAPDH to generate the column chart. *p* < 0.05 was marked as *, *p* < 0.01 was marked as **, *p* < 0.001 was marked as ***.

To explore the role of miR-373 as well as its regulation in OA, we first conducted qRT-PCR was used to determine its expression in OA patients. We found that miR-373 was decreased by more than 50% in OA samples (Figure 3A, *p* < 0.05). To test the interaction between CREB and miR-373, CHIP assay was done by incubating cloned miR-373 promoter fragments in immunoprecipitated CREB–chromatin complexes. As a result, the promoter fragment was effectively pulled down by the CREB-specific antibody after the incubation with chondrocyte lysate (Figure 3B). To confirm the binding specificity of CREB onto miR-373 promoter, we constructed three different mutant promoters (Mut 1-3; Supplementary Table S1) for dual-luciferase reporter assay. It showed that all mutations exhibited weaker luciferase activity than wild type to different extents (Figure 3C, *p* < 0.05 or *p* < 0.01). Next, we examined the effects of CREB overexpression/knockdown on the expression of miR-373 in chondrocytes. The results showed that CREB overexpression induced the expression of miR-373, while CREB knockdown repressed its expression (Figure 3D). Then, we used a miR-373 inhibitor to prove its role in the CREB-rescued OA *in vitro* model. We found that CREB overexpression significantly reduced TBHP-caused chondrocyte apoptosis indicated by TUNEL staining. During the TBHP treatment, transfected inhibitor NC had no impact on the reduced TUNEL counting but miR-373 inhibitor transfection led to a significant increase of chondrocyte apoptosis (Figure 3E). A similar reversive effect by miR-373 inhibitor was also observed in the aspects of autophagy flux and related proteins. In detail, the CREB-enhanced autophagosome (LC3B signal, Figure 3F) and lysosome activity (LysoTracker signal, Figure 3G) under TBHP treatment were reversed by miR-373 inhibition. Likewise, the cleaved Caspase-3, Bax, Bcl-2, and P62 were decreased while LC3II, LAMP2, and CTSB were increased in CREB overexpressed groups, and this function can be inversed by co-transfection with miR-373 inhibitor (Figure 3H). These results proved that CREB cannot rescue the survival and activity of chondrocytes under oxidative pressure partially without miR-373.

### METTL3 is a Direct Target of miR-373

**FIGURE 4:**
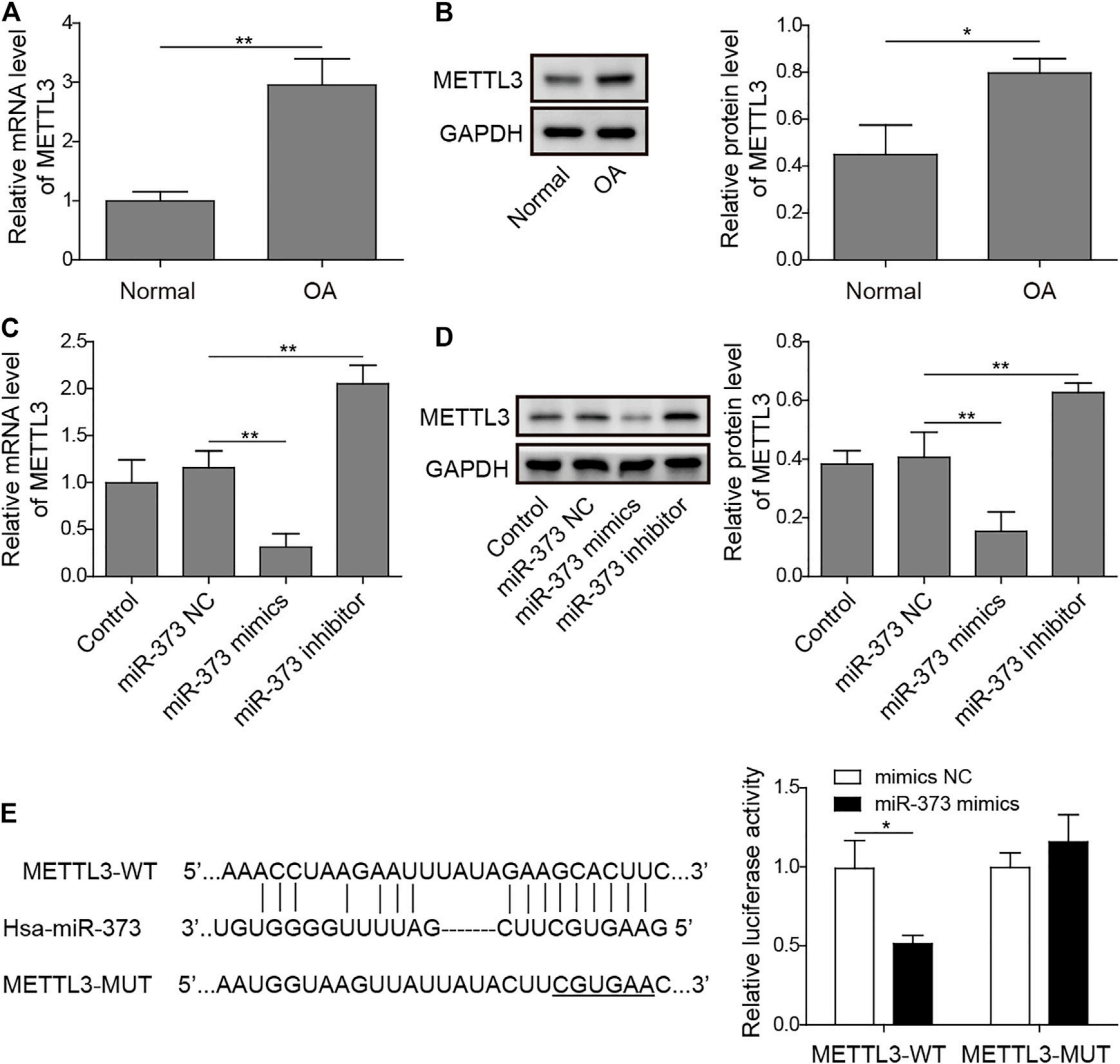
METTL3 was a direct target of miR-373. **(A,B)** The expression of METTL3 mRNA and protein in normal and OA samples was determined by qRT-PCR and Western blotting. GAPDH was used as the endogenous reference for normalization. **(C,D)** The effect of miR-373 mimic or inhibitor on the expression of METTL3 mRNA and protein in cultured human chondrocytes was determined by qPCR and Western blotting. GAPDH was used as the endogenous reference for normalization. **(E)** The targeting specificity of miR-373 to METTL3 was verified by dual-luciferase reporter assay. A miR-373-resistant METTL3 was generated as the shown sequence. Luciferase activity based on either METTL3-WT or METTL3-MUT was normalized to the corresponding mimic NC control separately. *p* < 0.05 was marked as *, *p* < 0.01 was marked as **.

By analyzing the target information of miR-373 from StarBase, a miRNA–mRNA interaction prediction database (Yang et al., 2011), we found that METTL3 turned out to be one of the prior candidates. Both mRNA and protein expressions of METTL3 were significantly increased in OA (Figures 4A,B). More than that, treatment of miR-373 mimic onto chondrocytes caused a significant decrease of METTL3 mRNA and protein while miR-373 inhibitor promoted its expression even higher than control/NC (Figures 4C,D). To further confirm the interaction between METTL3 and miR-373, the predicted miR-373-recognized sequence on METTL3 was mutated at 16 bases so that this mutation (MUT) should be resistant to the miRNA. Then, both WT- and MUT-METTL3 were fused to a fluorescence reporter for luciferase assay. Indeed, luciferase activity tagged with WT was dramatically reduced by miR-373 mimic but that of MUT was almost intact (Figure 4E), indicating METTL3 is a direct target of miR-373.

### METTL3 is Part of the CREB/miR-373 Axis Regulating the Autophagic Flux in Chondrocyte

**FIGURE 5:**
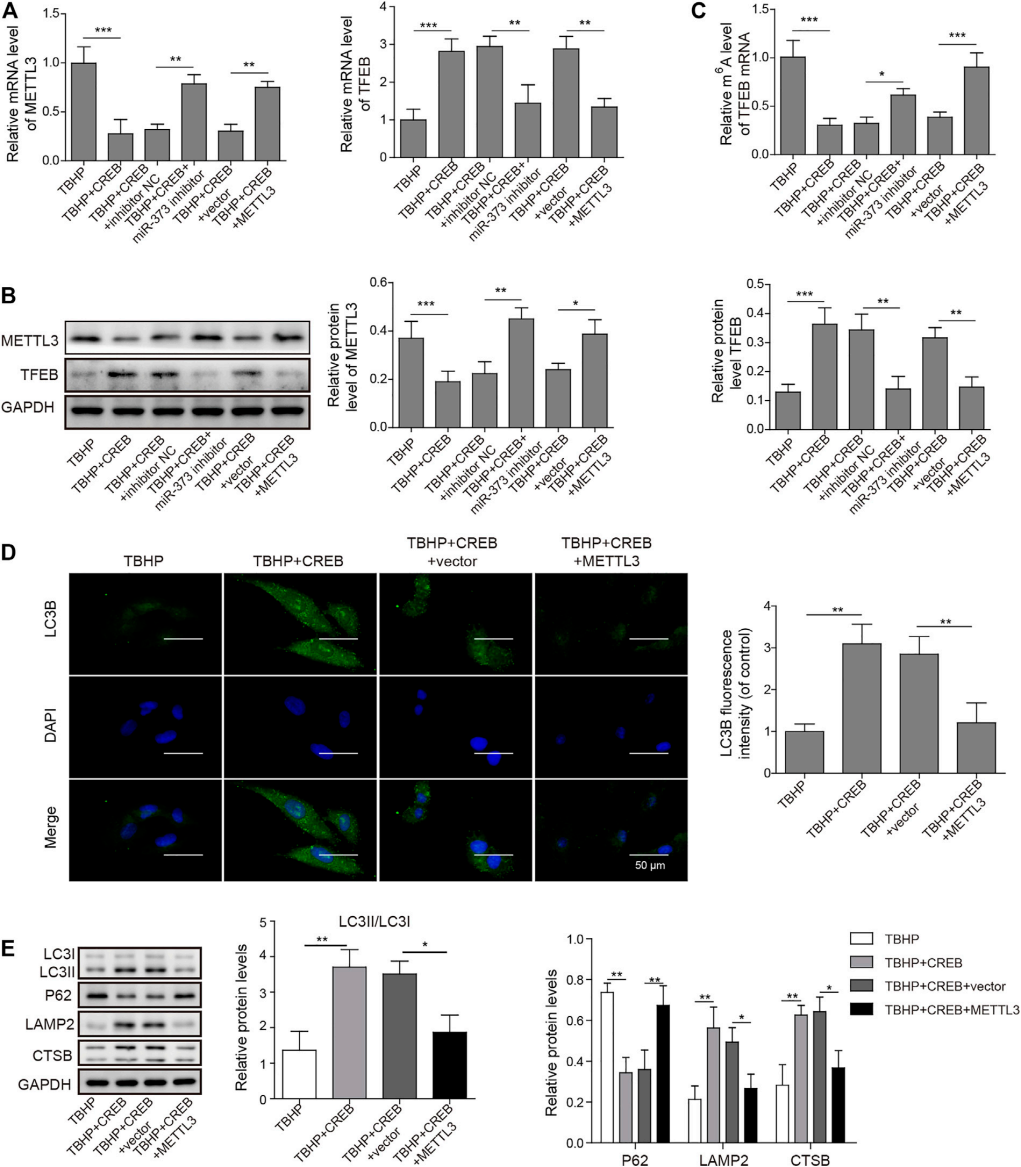
Overexpression of METTL3 overturned the CREB-promoted autophagic flux. **(A)** The expression of METTL3 (left) and TFEB (right) were confirmed by qRT-PCR. GAPDH was used as an endogenous reference. **(B)** The changes of the protein of METTL3 and TFEB in the experiment above were determined by Western blotting. Quantified intensity of the bands was normalized to GAPDH to generate the column chart. **(C)** The changes of m6A of TFEB mRNA in different treatments above. **(D)** The impact on the autophagic flux was determined by the immuno-staining of LC3B. LC3B fluorescence intensity was measured and data from all groups were normalized to the TBHP group. **(E)** The changes of the essential regulators or markers of apoptosis and autolysosome were determined by Western blotting. Quantified intensity of the bands was normalized to GAPDH to generate the column chart. *p* < 0.05 was marked as *, *p* < 0.01 was marked as **, *p* < 0.001 was marked as ***.

**FIGURE 6:**
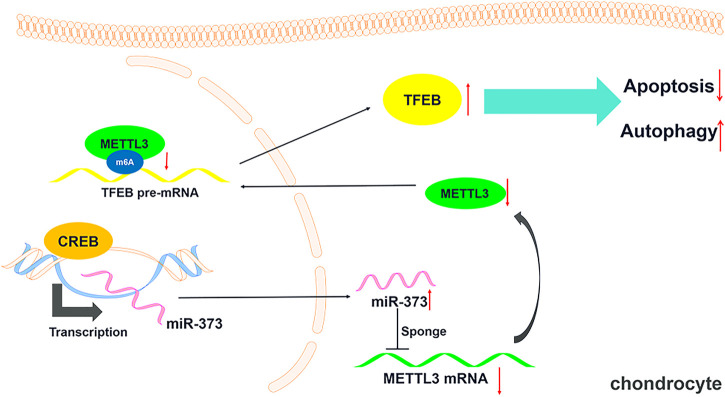
Schematic diagram of this study. A schematic model showing that CREB regulating chondrocytes autophagy/apoptosis *via* the miR-373/METTL3/TFEB axis.

To prove the role of METTL3 in OA development and the CREB-regulated pathway, we examined its change in the TBHP-induced OA cell model. We found that the overexpression of CREB significantly down-regulated METTL3. However, the downregulation of METTL3 by CREB was reversed by transfection of miR-373 inhibitor (Figure 5A Left). Together with the CREB-induced miR-373 increase result, it further proved that METTL3 was a part of the CREB-miR-373 regulation axis. We found that the autophagic flux master regulator TFEB was increased with the overexpression of CREB but decreased when miR-373 inhibitor co-transfection. Similarly, METTL3 overexpression also effectively suppressed the CREB-induced TFEB expression (Figure 5A Right). At the protein level, the changes of METTL3 and TFEB in chondrocytes under different treatments were consistent with their mRNA change (Figure 5B). This inverse correlation between TFEB and METTL3 inspired us to check the epigenetic changes on TFEB. As shown in the m6A quantification result, overexpression of CREB caused a decrease of m6A modification on the mRNA of TFEB (Figure 5C), which was coincident with the decrease of METTL3 expression and a consequent increase of TFEB expression itself (Figure 5A). The miR-373 inhibitor transfection under CREB overexpression led to an increase of TFEB m6A modification. Co-transfection of METTL3 and CREB overexpression vector increased TFEB m6A level as compared with CREB overexpressed chondrocytes. The changes of TFEB m6A level further proved that TFEB expression was regulated through CREB/miR-373 axis by METTL3’s epigenetic regulation. To confirm the effect exerted by this possible regulation axis, we examined the change of autophagic flux indicated by LC3B. Both immunostaining image and the quantification analysis of LC3B fluorescence strength showed that the overexpression of METTL3 dramatically suppressed the increase LC3B signal by CREB overexpression (Figure 5D). More than that, as revealed by Western blotting, CREB significantly increased LC3II, LAMP2, and CTSB but decreased P62, indicating a rescued autolysosomal function. However, the overexpression of METTL3 cut back the amount of these positive regulators and recovered P62 to the level as TBHP group (Figure 5E). Taken together, these results implied that the CREB/miR-373 axis-regulated autophagic flux in chondrocytes was partially by METTL3-mediated m6A modification of TFEB (Figure 6).

## Discussion

OA has long been considered a challenge to control due to its complex pathogenesis. Our study of this disorder has revealed a regulation axis initiated by CREB towards the TFEB-regulated chondrocyte autophagy in OA cartilage. We first found that CREB was significantly lower in clinic OA human cartilage samples than normal samples. A similar change was also found in the DMM mouse OA model. More than that, the overexpression of CREB in TBHP-treated human chondrocytes effectively attenuated the TBHP-induced apoptosis and the blockage of autophagic flux. This effect was proved to be dependent on miR-373, which is one of the most decreased miRNAs in OA cartilage (Cong et al., 2017). Next, we proved that METTL3 was targeted by miR-373 while this epigenetic regulator strictly controlled one of the most essential autophagy regulators TFEB in cartilage tissues. Taken together, we found a novel regulation axis initiated by CREB. This axis maintains the autophagy activity of chondrocytes to support their survival. This discovery may provide a novel ideal in both the understanding of the pathogenesis and the development of the treatment for this disorder.

As one of the most classic regulation pathways, CREB is involved in a tremendous number of biological processes. The change of CREB in OA has been mentioned in some studies and controversy also exists. In most of the studies, the result is consistent with our finding in which CREB is downregulated in either protein or phosphorylation modification in OA (Wang et al., 2017a; Sun et al., 2017). However, some show an increase in phosphorylated CREB (Ji et al., 2019). It is understandable because of the heterogeneous etiologic factors for this disorder. A different model may involve different interruptions in different networks. It reminds us that the underlying mechanisms are much more complex than imagined. Other than that, the alternation of CREB expression has always been regarded as an inevitable consequence rather than a start point. However, in our study, we for the first time provided solid evidence in which artificially increased CREB level in OA was able to protect the chondrocytes from loss of activity and even cell death. This not only proved that CREB could manage the development as a major regulator independently but also provided a potential therapeutic target.

Furthermore, although the decreased miR-373 in chondrocytes of OA has been continuously noticed (Song et al., 2015; Jin et al., 2017; Zhang et al., 2018) and long non-coding RNA PART1 has been proved to be a post-transcriptional regulator of miR-373 in chondrocytes (Zhu and Jiang, 2019), the direct upstream regulator is still unknown. In our study, we first confirmed that the expression of miR-373 was indeed decreased in both human samples and surgically induced mouse OA model, which again implied the possible engagement of miR-373 in OA etiology. More than that, as an answer to what the direct regulator of miR-373 on the transcription level is, our findings proved that CREB increases miR-373 expression by directly binding to its promoter. Interestingly, this interaction has also been observed in pancreatic cancer (Zhang et al., 2013), which not only shows the essential role of autophagy regulation in human health but also implies that the regulation axis starting from CREB may serve as one of the top candidates for the treatments to other diseases. Meanwhile, it has been discovered that the introduction of miR-373 into mice DMM model significantly reduced cartilage destruction (Song et al., 2015), which is also consistent with our results. Based on this discovery, we proved that the protection of chondrocyte autophagy from CREB depended on miR-373 induction. This discovery first provided another line of evidence supporting the essential role of non-coding RNA regulation, especially miRNA, in OA development as well as other osteopathological process (Wang et al., 2017b; Liu et al., 2017). Other than that, with the discovery of diverse transportation of miRNAs among cells such as the delivery by exosome (Li J. et al., 2017), the miRNA-conducted regulation within, or even beyond, specific cell type would also be of interest and necessity. In addition, the deepened understanding of non-coding RNA’s role in the development of OA may provide several novel diagnostic or therapeutic targets to strengthen the arsenal against bone-related diseases (Li Z. et al., 2017).

In addition to that, METTL3 was first identified as a direct target for miR-373. METTL3 is a key factor in a methyltransferase complex catalyzing the modification of N^6^-methyladenosine (Liu et al., 2014). This modification is the most prevalent epigenetic modification of mRNA (Zhao et al., 2016). The role of METTL3 on OA etiology has been discussed recently. It has been reported that the expression of METTL3 was elevated in chondroprogenitor cells after IL1-β stimulation, and the consequent m6A increase resulted in OA development through severe inflammatory and extracellular matrix degradation. Similarly, our research found that the overexpression of METTL3 promoted OA-like phenotype through the inhibition of autophagy flux. More importantly, we identified TFEB as one of the METTL3 targets, which was responsible for the effect of METTL3 in OA development. This is inspiring not only because TFEB was recently proved to be a target of METTL3 in cardiomyocytes (Song et al., 2019), but also because it is widely regarded as a core regulator in autophagic and lysosomal biogenesis (Settembre et al., 2011; Decressac et al., 2013; Lapierre et al., 2013). Moreover, TFEB has been reported to control autophagy of the endoplasmic reticulum in chondrocytes by inducing the expression of FAM134B, an endoplasmic reticulum autophagy receptor (Cinque et al., 2020), which might explain its beneficial effects on OA. Taken together, these findings integrate a major autophagy regulator into an epigenetic network, and it further consolidates the position of METTL3 in OA development and autophagy regulation within the condition. Besides, this may serve as a novel target for a more effective treatment. More interestingly, it should be emphasized that the pathway being studied in the previous and present research, either downstream or upstream, is diverse, implying that a regulation network centered by CREB/miR-373/METTL3 is still to be further unveiled.

In summary, our studies demonstrate that CREB plays a critical role in the pathogenesis of OA. As a transcription factor, it controls the expression of miR-373, which directly targets an important epigenetic regulator METTL3 and finally relieves TFEB from the METTL3-conducted epigenetic suppression. This regulation axis may serve as a novel target for the treatment of OA.

## Data Availability

The original contributions presented in the study are included in the article/Supplementary Material, further inquiries can be directed to the corresponding author.

## Abbreviations

CREB, cAMP response element-binding protein; CQ, chloroquine diphosphate; CSTB, cystatin B; LC3B, microtubule-associated proteins 1A/1B light chain 3B; LAMP2, lysosome-associated membrane protein-2; METTL3, methyltransferase-like 3; OA, Osteoarthritis; ULK1, Unc-51–like kinase 1; PDGF-BB, platelet-derived growth factor BB; TBHP, tert-butyl hydroperoxide.
