# Supplementary material for: CREB Ameliorates Osteoarthritis Progression Through Regulating Chondrocytes Autophagy via the miR-373/METTL3/TFEB Axis
Source: Front Cell Dev Biol. 2022 Jun 9;9:778941. doi: 10.3389/fcell.2021.778941 (PMC9218638; doi:10.3389/fcell.2021.778941)
Supplement: Supplementary file 1 [file Table1.DOCX]

Supplementary Table 1

The primer of miR-373 mutant promoters

| Primer | Mutation Type | Sequence 5'-3' |
| --- | --- | --- |
| Mut-1-F | CAGTGATGGCAGA | GTCAGTCACTACCGTCTTCCTCGCGAGGAGCTCATAC |
| Mut-1-R |  | GAAGACGGTAGTGACTGACTTTCTTGCCTACAAGAGGTTGG |
| Mut-2-F | ACCGGTGACGCC | GTCTGGCCACTGCGGCATATCAACGGATGCCGTGGAG |
| Mut-2-R |  | ATGCCGCAGTGGCCAGACGCTCAAATGTCGCAGCA |
| Mut-3-F | GGTGTGGACGTA | GACCACACCTGCATTCATTGGCCTCTGTGAGTATTGCT |
| Mut-3-R |  | ATGAATGCAGGTGTGGTCAGGTCTGCCAGAAAGAACAAGG |
